# Use of a mobile application for Ebola contact tracing and monitoring in northern Sierra Leone: a proof-of-concept study

**DOI:** 10.1186/s12879-019-4354-z

**Published:** 2019-09-18

**Authors:** Lisa O. Danquah, Nadia Hasham, Matthew MacFarlane, Fatu E. Conteh, Fatoma Momoh, Andrew A. Tedesco, Amara Jambai, David A. Ross, Helen A. Weiss

**Affiliations:** 1 School of Public Health, Faculty of Medicine, Imperial College London, London, UK; 2 MRC Tropical Epidemiology Group, Faculty of Epidemiology and Population Health, London School of Hygiene & Tropical Medicine, London, UK; 3 Innovations for Poverty Action, Freetown, Sierra Leone; 4 Ministry of Health and Sanitation, Freetown, Sierra Leone

**Keywords:** Ebola, Contact tracing, Sierra Leone, mHealth

## Abstract

**Background:**

The 2014–2016 Ebola epidemic in West Africa was the largest Ebola epidemic to date. Contact tracing was a core surveillance activity. Challenges with paper-based contact tracing systems include incomplete identification of contacts, delays in communication and response, loss of contact lists, inadequate data collection and transcription errors.

The aim of this study was to design and evaluate an electronic system for tracing contacts of Ebola cases in Port Loko District, Sierra Leone, and to compare this with the existing paper-based system. The electronic system featured data capture using a smartphone application, linked to an alert system to notify the District Ebola Response Centre of symptomatic contacts.

**Methods:**

The intervention was a customised three-tier smartphone application developed using Dimagi’s CommCare platform known as the Ebola Contact Tracing application (ECT app). Eligible study participants were all 26 Contact Tracing Coordinators (CTCs) and 86 Contact Tracers (CTs) working in the 11 Chiefdoms of Port Loko District during the study period (April–August 2015). Case detection was from 13th April to 17th July 2015. The CTCs and their CTs were provided with smartphones installed with the ECT app which was used to conduct contact tracing activities. Completeness and timeliness of contact tracing using the app were compared with data from April 13th-June 7th 2015, when the standard paper-based system was used.

**Results:**

For 25 laboratory-confirmed cases for whom paper-based contact tracing was conducted, data for only 39% of 408 contacts were returned to the District, and data were often incomplete. For 16 cases for whom app-based contact tracing was conducted, 63% of 556 contacts were recorded as having been visited on the app, and the median recorded duration from case confirmation to first contact visit was 70 h.

**Conclusion:**

There were considerable challenges to conducting high-quality contact tracing in this setting using either the paper-based or the app-based system. However, the study demonstrated that it was possible to implement mobile health (mHealth) in this emergency setting. The app had the benefits of improved data completeness, storage and accuracy, but the challenges of using an app in this setting and epidemic context were substantial.

**Electronic supplementary material:**

The online version of this article (10.1186/s12879-019-4354-z) contains supplementary material, which is available to authorized users.

## Background

The 2014–2016 Ebola virus disease (EVD) (Ebola) epidemic in Liberia, Guinea and Sierra Leone resulted in over 28,000 reported cases and over 11,000 deaths [1]. Sierra Leone was the most severely affected country, with 8706 laboratory confirmed cases and 3956 deaths [1]. During the epidemic, surveillance goals were to detect Ebola cases promptly to provide an effective response including rapid diagnosis, case isolation and management, contact tracing, safe burials, and identification of transmission chains [2, 3]. Contact tracing for Ebola is defined as the identification and follow-up over 21 days of individuals who have been in contact with a person confirmed to have been infected with Ebola [2]. Previous epidemics have been controlled through contact tracing and isolation, which limits onward transmission [4].

Contact tracing was a core surveillance activity in the 2014–2016 epidemic [5, 6]. Ebola contact tracing operations used paper-based systems supplemented by phone calls and Short Message Service (SMS). Challenges identified in previous epidemics include incomplete identification of contacts and inefficiencies in paper-based reporting systems, including missing contact lists, inadequate data collection and training, transcription errors and delays in steps from identification of contacts to isolation of suspected cases among those contacts [2, 5, 7–10].

The national system for contact tracing in Sierra Leone during the 2014–2016 Ebola epidemic was that, after the initial alert of a suspected or probable case, a case investigation team (CIT) investigated to determine whether the person was a suspected or probable Ebola case. If the case definition was met, the CIT conducted an initial line listing of contacts of the case. The case (suspected or probable) was then escorted to an Ebola Treatment Centre (ETC) or holding centre. If laboratory-confirmed as EVD, contact tracing was initiated, using a paper Daily Reporting Form (DRF). Challenges specific to contact tracing during the 2014–2016 Ebola epidemic included insufficient trained staff, community resistance to report Ebola cases and contacts and mistrust of contact tracers, limited telephone and internet connection, difficulties in reaching remote areas and the high number of Ebola cases that occurred within a short period [5, 9]. Due to these challenges, we established a study to assess the ability of an mHealth app to improve contact tracing through using mobile phones.

### mHealth approaches for epidemic investigations

Use of mobile devices to support medical and public health practice (mHealth) can improve health outcomes in low-income settings [11–14], due to the low cost of roll-out, mobility of devices, ease of use and flexible deployment compared with other methods e.g. computers. mHealth applications have the potential to reach rural populations with low levels of income and literacy, and to reduce time to collect data, distance travelled to collect and return information and the cost of information delivery [11, 12, 15, 16]. mHealth applications have been used for epidemic tracking of tuberculosis (TB) and Japanese encephalitis, but few mHealth applications have been designed and evaluated for contact tracing. One app designed for contact tracing for TB in Botswana reduced the median time required to complete the TB contact tracing form and improved data quality compared to paper-based contact tracing [17]. However, the full potential of mHealth in addressing disease epidemics is largely unexplored.

During the 2014–2016 Ebola epidemic, several mHealth interventions were developed to improve patient care, health worker safety, public awareness, surveillance and vaccine monitoring [18–26]. Studies have reported on the use of mobile phones for community event-based surveillance through reporting of sick community members with symptoms suggestive of Ebola to community and district monitors through audio phone calls and SMS messages to support surveillance initiatives [20, 27]. One of the few apps for Ebola contact tracing was developed by the Earth Institute at Columbia University. This consisted of a smartphone application developed using the CommCare application for collecting and analysing contact tracing data in Guinea [10]. The system demonstrated the potential to improve data access and improve the quality of surveillance data.

### Evaluating mHealth interventions

Most studies of mHealth applications are small-scale or pilot implementation studies, with few rigorously designed evaluation studies [28–30]. This is likely due to inherent challenges in introducing technologies in complex emergencies, including the feasibility of implementing technology during an ongoing epidemic [10] .

#### Objective

Our hypothesis was that the mHealth system would be more effective for contact tracing than the standard paper-based system. The original aims were to i) evaluate the feasibility of introducing a smartphone-based electronic data-capture and management system, through the development of a smartphone app for Ebola contact tracing and monitoring in Port Loko District, Sierra Leone, and ii) compare the effectiveness of the app with the standard paper-based system of contact tracing through a cluster randomised trial. The objectives were to i) assess the time between a new Ebola case being laboratory-confirmed and their household contacts first being visited by a contact tracer, and ii) evaluate the operational requirements needed to introduce effective mobile-based contact tracing and monitoring in Sierra Leone.

Due to the decline in Ebola cases in Port Loko District during the first 6 months of 2015, the study design was changed on the June 8, 2015 to a proof-of-concept (demonstration) project. The study aim was revised to describe the feasibility and effectiveness of an electronic data capture and management system to improve monitoring of contacts of confirmed Ebola cases, and to provide guidance on how best to implement the system. The objectives remained unchanged.

The objective of this manuscript is to describe the results of this proof-of-concept study to assess the feasibility and effectiveness of implementing the electronic data capature and management system for contact tracing through comparison with the standard paper-based system for contact tracing.

## Methods

### Setting and participants

The study was conducted in Port Loko District, northern Sierra Leone, and was a collaboration between the London School of Hygiene & Tropical Medicine (LSHTM), Innovations for Poverty Action (IPA) and International Medical Corps (IMC), working closely with the District and National Ebola Response Teams. In Port Loko District, the overall technical supervision of surveillance and contact tracing activities was managed by the World Health Organization (WHO). The lead agency for the management and supervision of surveillance activities including contact tracing activities was Marie Stopes International until the end of March 2015, and was taken over by GOAL Global on April 1st 2015. UNFPA were responsible for the management and supervision of contact tracers.

Eligible study participants were all 26 Contact Tracing Coordinators (CTCs) and 86 Contact Tracers (CTs) operating in the District during the study period (April 13 to August 31, 2015). The list of eligible CTCs and CTs was obtained from partners responsible for contact tracing activities in the District.

### Recruitment and informed consent

Written informed consent was obtained from eligible CTCs and CTs who consented to take part in the study. Consent was not required from individual Ebola contacts as the smartphone app mirrored the existing paper-based system. The only additional information collected was to record the time of the first contact tracing visit.

### Study definition - Ebola contact

The study definition of an Ebola contact was that used by the Sierra Leone National Emergency Response Centre, i.e. a person without any disease signs and symptoms who had physical contact with an Ebola case (alive or dead) or the body fluids of an Ebola case within the last three weeks. Physical contact included sharing the same room/bed, caring for a patient, touching body fluids, or closely participating in a burial [31]. An Ebola contact was defined as a listed^1^ contact of a laboratory-confirmed Ebola case in Port Loko District diagnosed during the study period.

The line listing of Ebola contacts was undertaken using the Ministry of Health and Sanitation, Ebola Virus Disease Contact Line Listing form. This was done by the CIT and involved asking household members of the Ebola case to report on the individuals that the Ebola case had come into contact with that met the definition used by the Sierra Leone National Emergency Response Centre. Data on each person who had contact with the Ebola case (Ebola contacts) was collected, including their name, demographic and location information, date of last contact with the Ebola case, type of contact, head of household and contact details. The initial line list was then updated once the suspected or probable Ebola case was confirmed by the laboratory. Once the Ebola case was confirmed, contact tracing of the line listed contacts started.

### Original study design

**Fig. 1:**
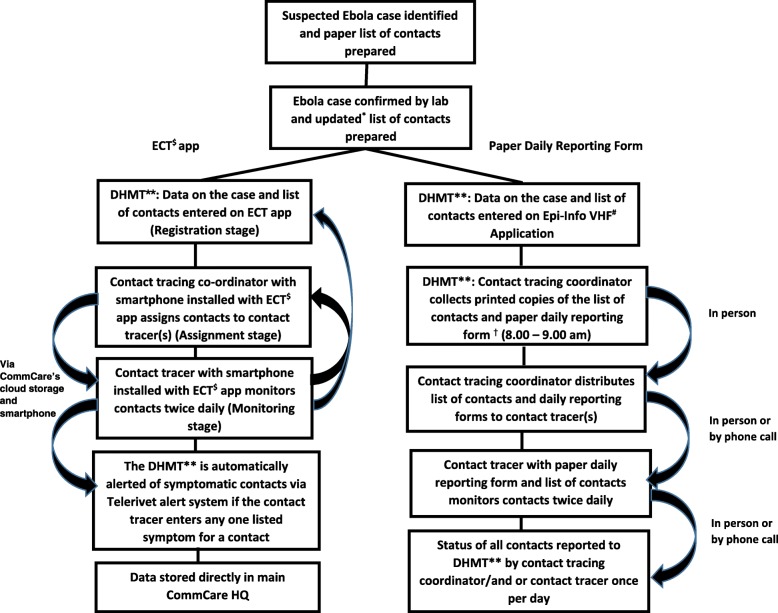
Design of the Ebola Contact Tracing smartphone app study. Key. * The surveillance team goes back to the house of the suspected case to obtain an updated list of contacts following laboratory confirmation. ** District Health Management Team. ^#^ Viral Hemorrhagic Fever. ^†^ Contact Tracing Coordinators visit the DHMT six days a week (Monday to Saturday). ^$^ Ebola Contact Tracing

The original design was a two-arm cluster-randomised trial to be conducted in all 11 Chiefdoms of Port Loko District. The Chiefdoms were randomised in a 5:6 allocation ratio to receive one of the following: i) Control arm: standard paper-based contact tracing system, involving CTCs operating at Chiefdom level within the District, together with a team of CTs based in villages throughout the Chiefdom using a paper-based system supplemented by ad-hoc phone calls and SMS messaging for contact tracing of contacts of confirmed Ebola cases ii) Intervention arm: the CTCs and their CTs were provided with smartphones installed with the Ebola Contact Tracing (ECT) app (Fig. 1). CTCs and CTs were trained and supported to use this system. The trial started on April 13, 2015, with the intervention introduced in 6 randomly selected Chiefdoms and ongoing paper-based data collection in the remaining 5 Chiefdoms.

### Modified study design

Due to the small number of Ebola cases, the design was changed on June 8, 2015 to a proof-of-concept study in order to introduce the app to all 11 Chiefdoms in the District. This decision was taken to maximise the data collected using the app given the small number of new Ebola cases and that these had occurred within a short period of time. Following the recommendation of local WHO staff, the paper-based system for contact tracing continued to be used alongside the ECT app as it was the standard national system and was considered essential for surveillance. The date and time of the Ebola confirmation for all cases was from the Public Health England (PHE) laboratory in Port Loko District.

### Qualitative methods

Qualitative methods formed part of a process evaluation to evaluate the intervention. This included semi-structured interviews and focus group discussions (FGDs) with district-level data entry and management staff, CTCs and CTs on their use of mobile technology prior to the study, use of the smartphone app for contact tracing, training and ideas for improvements to the app. Semi-structured interview schedules and a topic guide were developed that addressed key areas in relation to the study objectives, study operation, implementation and delivery. The semi-structured interviews and focus group discussions were conducted in either English or Krio and were audio recorded.

### Development and testing of the Ebola contact tracing (ECT) app

The intervention (“ECT app”) was a customised three-tier smartphone application developed using Dimagi’s CommCare platform [32], an open-source mobile platform for electronic data capture and case management. It was developed from February–April 2015 by a developer in the USA (SQ) working with the study team. The primary reasons for using CommCare were i) its case management feature, which enables information for an index Ebola case to be linked to multiple contacts per case; and ii) the ability to share data on the Ebola cases and their contacts between multiple users (e.g. CTCs, CTs and the study team). The app was pre- and pilot tested, and refined iteratively before deployment in mid-April 2015. Due to the timeline, app structure and feasibility of making technical changes, not all required changes could be made to the app, however, mitigating action was taken to overcome these issues (Additional file 1: Table S1). The smartphones were second-hand Android phones (4G HTC MyTouch) donated by the UN Mission for Ebola Emergency Response (UNMEER).

The app was developed following an assessment of the structure of the contact tracing system in the District, and discussions with national and local stakeholders including the National Emergency Response Centre Surveillance Pillar, the WHO Country Office, the Port Loko District Health Management Team (DHMT), stakeholders from the DERC, District Council, US Centers for Disease Control, WHO (Port Loko), Marie Stopes International, UNFPA and GOAL Global (Additional file 2).

### Phased introduction of the ECT app

A phased introduction of the intervention (the ECT app) was formally approved by the Sierra Leone Ministry of Health and Sanitation and the WHO (Freetown national office) on February 9, 2015. This involved pilot testing the intervention in one ‘low risk’ Chiefdom (with no confirmed Ebola cases within the previous 21 days) from March 5–10, comprising a 2-day training workshop and a 4-day simulation exercise. Following feedback and revisions to the app, a second pilot test was conducted from March 16–18. The results were presented to stakeholders, and authorisation was granted to phase in the intervention.

### Study training

The main study training was on April 14–24, 2015 for all 26 CTCs from the 11 Chiefdoms. This included an initial half-day of training on the study design and use of the paper DRF for contact tracing. Those in the intervention arm Chiefdoms had a further 2.5 days training after the initial training on use of the smartphone and ECT app. This training was prior to the modified design of a proof-of-concept study. Training for CTCs from the control arm Chiefdoms subsequently included in the proof-of-concept study took place from June 9–20. Prior to this training, the paper-based system was the sole contact tracing system in the control Chiefdoms.

### Data analysis

The data collected via the app was stored on a central server on CommCareHQ and exported into Excel and checked before being transferred to Stata version 14 for data management. Data from the paper-based DRFs were entered into Excel, and exported to Stata for data management. Descriptive analyses were used to estimate the duration from laboratory confirmation of an Ebola case to time of first visit to any one of their contacts monitored using the app or paper, and the median duration of each step of the chain (confirmation of Ebola case to Ebola case registration, to Ebola contact assignment by CTCs using the app, to first visit by the CT; Table 1). Results were summarised by case, and also over all the contacts to give equal weight to each contact, as the number of contacts per case varied substantially.

**Table 1:** Features and functionality of the Ebola Contact Tracing (ECT) App

ECT Module	Purpose	System features and advantages over paper-based system
Registration by Data Co-ordinator at District level	Registration of data for confirmed Ebola case and all listed contacts	• Built-in validation features (improves data accuracy e.g. unique ID, number of digits for phone numbers and mandatory fields e.g. age, sex) • Skip logic and mandatory data entry ensures only relevant questions are answered • Automatic repeat information features (e.g. same address details of case and contacts) reduces time • Additional contacts feature (ease of adding additional contacts to app saves time • Password-secured (ensures confidentiality of Ebola cases and contacts) • District level monitoring (improve monitoring at district level)
Contact assignment by Contact Tracing Co-ordinator at Chiefdom level	Assignment of listed contacts by CTC to a particular Contact Tracer (CT) in a Chiefdom (CTC is geographically mobile and can assign from anywhere where there is an active data connection)	• Real-time data transmission to CT (reduces interval before contact monitoring) • Limited contact information view (View only contacts for a specific Chiefdom improves confidentiality and accuracy • Named CT assignment feature (reduces chances of assignment error) • Reassignment feature (enables contacts to be easily re-assigned if misassigned) • Pre-loaded contact information (minimises errors as no further data entry required) • Swipe system for contact assignment (minimises reliance on typing/ reduce data entry error) • Multiple contact assignment feature (enables contacts to be assigned to one or more CTs)
Visitation by Contact Tracer (CT) at community level	To enable the CT to monitor the listed contacts assigned to them for a 21 day period or until the contact becomes symptomatic	• Real-time information transmission (reduces time-lag for the start of contact monitoring) • Pre-loaded information on assigned contacts received (eliminates data re-entry/reduces data entry errors) • Automatic GPS location tracker (enables monitoring of where data entry on the app took place) • Automatic date and time capture (enables monitoring of CT performance and reduces data misreporting) • Offline data entry (mobile platform permits offline data entry – contact monitoring can be entered offline/reduces reliance on good network coverage) • Repeat visits information can be entered (repeat contact visit information can be entered and automatically uploaded with immediate data storage once synced) • Password secured data (ensures confidentiality and data security) • Additional data feature (e.g. on food availability) can be sent to the district for improved care and coordination.
Telerivet Alert Line	To alert the district Ebola Response Centre of symptomatic contacts identified by the CT during the contact visitation step	• Real-time district alert of symptomatic contacts (enables quick dispatch of surveillance team and potentially reducing the time to isolation • Password secured data (ensures confidentiality) • Reduced reliance on phone calls or SMS messages (ensures all symptomatic contacts are accurately logged)

Key: CTC Contact Tracing Coordinator; CT Contact Tracer; GPS Global Positioning System; SD Secure Digital; SIM Subscriber Identity Card; SL Sierra Leone; SMS Short Message Service

Qualitative data were translated and transcribed into English. All data were anonymised and any identifying information was removed prior to the analysis. The data was then analysed using thematic analysis to identify, analyse and report patterns (themes) from the data using NVivo software [33]. A six-step approach was undertaken for the thematic analysis as described by Braun and Clarke (2006) [33].

### Ethics

Ethics approval was received from The London School of Hygiene & Tropical Medicine Observational/Interventions Research Ethics Committee (reference 8749–01) and the Sierra Leone Ethics and Scientific Review Committee (SLESRC). The study received clearance and support from the Republic of Sierra Leone Ministry of Health and Sanitation, and the National Ebola Response Centre Surveillance Pillar (responsible for Ebola contact tracing and monitoring activities nationally).

## Results

### Ebola cases

Of the 43 EVD cases identified during the study period, the contacts of 25 were monitored only using the standard paper-based system for contact tracing (“paper-based cases”). Contacts of the remaining 18 cases were also monitored on the ECT app (“app-based cases”). The cases were confirmed between the 13th April and 17th July 2015. The median age of paper-based and app-based cases was 26 years (interquartile range (IQR) 20–44) and 29 years (IQR 18–40), respectively (Table 2). Approximately half the cases were female (60% for the paper-based cases and 50% for the app-based cases; *p* = 0.52). All but one of the paper-based cases were from one Chiefdom. The 18 app-based cases were distributed across 5 Chiefdoms (Table 2).

**Table 2:** Characteristics of cases and contacts (13th April – August 31st 2015)

	Paper Daily Reporting Form		ECT Smartphone App	
	Cases	Contacts	Cases	Contacts
Characteristic	N (%)	N (%)	N (%)	N (%)
Total	25^a^	408	18^b^	646
Age				
< 5 yrs	4 (16%)	51 (13%)	2 (13%)	123 (19%)
5–14 yrs	2 (8%)	119 (29%)	2 (13%)	141 (22%)
15 – 44 yrs	13 (52%)	188 (47%)	9 (56%)	272 (42%)
≥ 45 yrs	6 (24%)	46 (11%)	3 (19%)	110 (17%)
Median (IQR)	26 (20–44)	18 (7–30)	28.5 (18–40)	20 (6–35)
Sex				
Male	10 (40%)	205 (50%)	9 (50%)	317 (49%)
Female	15 (60%)	203 (50%)	9 (50%)	329 (51%)
Mortality status				
Alive	21 (84%)	407^c^ (100%)	11 (61%)	646 (100%)
Dead	4 (16%)	–	7 (39%)	–
Chiefdom				
1	–	–	5 (28%)	72 (11%)
2	24 (96%)	376 (92%)	4 (22%)	70 (11%)
3	–	–	–	10^d^ (2%)
4	–	–	1 (6%)	65 (10%)
5	–	–	5 (28%)	159 (25%)
6	–	–	3 (17%)	270 (42%)
7	1 (4%)	32 (8%)	–	–

^a^Four cases followed using the paper Daily Reporting Form had no contacts

^b^ Data on age and sex were available for only 16 cases (not for two ‘secret burials’)

^c^ Data on 407 contacts was available. All contacts were alive

^d^The 10 contacts in this Chiefdom came from a case that occurred in Chiefdom 2

### Contacts of Ebola cases

A total of 408 contacts were identified for the 25 paper-based cases (“paper-based contacts”) (average 16 per case), and 646 contacts for the 18 app-based cases (“app-based contacts”) (average 36 per case). The age and sex distribution of contacts were similar by type of case (Table 2).

### Standard paper-based system for contact tracing

The paper DRF was returned for the contacts of only 9 of the 25 paper-based cases (36.0%) and for 157 of the 408 contacts of these cases (38.5%) at the end of the study period. The data collected were of poor quality, including errors on the forms (including missing or obviously incorrect time of first visit, Ebola case IDs and dates of first and last visit). A common error was that the time of contact visit reported by the CT on the DRF was reported as prior to the laboratory confirmation of the index case for 63 (40%) of the paper-based contacts. Among the remaining 94 contacts with completed forms, the median duration from laboratory confirmation of the Ebola case to first reported contact visit by the CT was 16.9 h (IQR 15.9–61.8 h). Fewer than half of the contacts (*n* = 43, 44%) were reported to have been monitored for the full 21 days.

### Influence of training on paper-based contact tracing

One Ebola case was confirmed the day before training started, and served as an example case during training. The quality of paper-based contact tracing data for this case was higher than for subsequent paper-based cases. For example, all forms for the 32 contacts of the case were returned, and 31 of these had time-of-first-visit data in line with the standard protocol of CTCs collecting the list of contacts from the DHMT between 8 and 9 am each morning and contact visits being made around midday. In contrast, for the 6 subsequent cases from another Chiefdom, 65 contacts were all recorded as having time-of-first-visit at 8.30 am (mainly on the same day as each other) which according to the standard protocol was not possible.

### App-based reporting for contact tracing

Of the 18 app-based cases, 2 were ‘secret burials’^2^ and were not laboratory-confirmed. For the 16 confirmed app-based cases entered on the ECT app for contact monitoring, 556 contacts were registered (Table 3). Of these, 524 contacts were assigned by CTCs (94.2%) for contact tracing, and 384 (62.5%) were visited. The 172 contacts who were not assigned or visited were from 5 different cases each from a different Chiefdom. The median time from laboratory confirmation to case registration on the ECT app was 18.0 h (IQR 12.1–30.0), from case registration to contact assignment by CTCs was a median of 23.4 h (IQR 3.5–46.1) and from median time of contact assignment to median time of first visit by a CT was 22.8 h (IQR 11.7–108.7; Table 3). Overall, the median time from laboratory confirmation to first recorded visit by the CT was 70.2 h (IQR 61.9–124.5) (equal weight per case). Key factors associated with the delays are described in Table 3.

**Table 3:** Time from laboratory confirmation to first visit for the 16 confirmed cases, plus two secret burials

Case	Chiefdom	Contacts registered (N)	Contacts visited (N)	Date/time of case confirmation	Case confirmation to registration (hours)	Case registration to contact assignment (median hours)	Contact assignment to first visit (median hours)	Case confirmation to first visit (median hours)	Case-specific delaying factors and comments
Total		556	384		18.0	23.4	22.8	70.2	
1	1	19	19	06/06/2015 19:23	19.99	29.73	22.77	72.56	● Remote location of contacts ● Limited network coverage ● Network coverage and syncing problems ● Battery charging issues
2	1	9	9	06/06/2015 22:18	43.89	3.16	23.12	70.16	
3	1	9	9	07/06/2015 06:27	9.25	3.58	49.07	61.89	
4	1	11	11	09/06/2015 23:10	16.52	1.68	21.77	39.94	
5	2	24	24	13/06/2015 15:20	27.13	46.17	26.69	99.74	● Remote Chiefdom ● Several cases occurring in close proximity and time to each other ● Technical difficulties assigning contacts^a^ ● Far from place where phones could be charged
6	2	14	14	17/06/2015 15:13	2.61	13.14	108.75	124.47	
7	2/3	36	34	18/06/2015 15:58	42.75	4.53	22.47	67.05	
8	2	6	6	24/06/2015 17:10	3.55	11.36	3.83	18.73	
9	4	24	15	29/06/2015 21:06	16.05	70.28	1.39	87.72	● Technical difficulties assigning contacts^a^ ● Transport difficulties for CTs led to CTC monitoring with paper form^b^
10	4	120	98	16/06/2015 22:54	11.43	81.05	128.26	251.97	
11	5	52	52	12/07/2015 14:26	19.57	2.88	5.35	27.81	● Long contact lists ● Contacts added after initial entry and assignment ● Long CT travel distances ● Management error in CT assignment^c^ ● WHO intervention including CTCs and CTs having to monitor additional non-quarantined households ^d^ ● Monitoring issues for CTs ^e^ ● Syncing and network connectivity difficulties ● Technical problems^f^ ● Misplaced phones Battery charging issues
12	5	23	23	14/07/2015 11:10	21.96	29.25	18.13	69.31	
13	5	5	5	17/07/2015 15:01	30.95	23.43	11.7	66.08	
14	6	112	51	25/06/2015 21:11	14.28	26.83	122.75	163.56	● CTC user error in assignment requiring retraining and support ● System errors with monitoring features. (Rectified for later cases)
15	6	92	14	02/07/2015 17:21	16.08	76.95	275	296.54	
16	5	0	0	18/06/2015 21:26	59.51				● No contacts registered
Secret burial 1	1	24	24			3.21	89.37		● Syncing delays or network problems ● Late reassignment of contacts
Secret burial 2	6	66	0			59.32			● Inaccessible due to flooding

Footnotes

^a^ This included difficulties in basic app functions including switching on mobile data to use the app, syncing difficulties to receive and send contacts

^b^ CTs experienced difficulties traveling to monitor contacts so the CTC monitored the contacts, but as the app on the CTC phone was for assignment and not monitoring, the CTC used the paper form for monitoring

^c^ CTCs often incorrectly assigned to a named Chiefdom level feature which showed on the page of named CTs to assign to. This was a technical feature of the app that could not be hidden which meant that the contact was not monitored by the correct CT

^d^ Extra responsibilities were placed on CTs in this Chiefdom to also conduct monitoring of non-quarantined homes due to a spike in cases in this Chiefdom. This was on the advice of WHO

^e^ This included transportation issues of CTs to monitor contacts, syncing issues, network connectivity difficulties and technical problems, misplaced phones, battery charging issues that were specific to this Chiefdom when there were cases

^f^ Technical problems related to the study phones included syncing issues to receive and send information to the CommCare server, software error messages, and corrupt SD card problems that caused the app to not work

Table 4 summarises results at contact level. The median duration from contact registration on the ECT app to assignment by CTCs was 4.33 h (IQR 1.4–47.5 h) but the median duration from contact assignment to first recorded visit by CTs was 24.9 h (IQR 18.1–122.7 h). The total median duration from the start of case registration on the ECT app at the district to the first recorded visit by the CT was 73.2 h (IQR 26.2–216.8 h) (equal weight per contact). The median number of days monitored for the 384 contacts was 10 days (IQR 5.5–16). This varied by Chiefdom with one Chiefdom having a median recorded follow-up of just 3 days (IQR 1–4).

**Table 4:** Duration of each step from case registration to first visit of each contact, using the ECT app

Step	N	Median	Min	25%	75%	Max
Time from case registration to contact assignment	556	25.6 h	0.1	0.5	30.6	147.2
Time from contact registration to contact assignment	524^a^	4.33 h	0.2	1.4	47.5	292.5
Time from contact assignment to first recorded visit	384^b^	24.9 h	0.4	18.1	122.7	304.3^c^
Total time from start of case registration to first recorded visit	384	73.2 h	4.6	26.2	216.8	384.9

Footnotes:

^a^ For 32 contacts, there was no record of assignment

^b^ For 140 contacts, there was no record of a visit at home

^c^ The 127 contacts with a duration of over 100 h were contacts of 4 cases. For one case, the long duration was due to syncing and network problems experienced by the CT. The SIM card had to be changed to a different network. For another, the CTC was not able to assign the contacts correctly as they found it difficult to assign the unusually large number of contacts and required assistance from the field team on the process. This led to contacts having to be reassigned. Specific field team training on monitoring assigned contacts in the CommCare HQ was not provided until July – after the major assignment problems had occurred

### Qualitative results

Results from the process evaluation indicated that few (< 5%) CTCs and CTs had previously used a smartphone but both CTCs and CTs preferred the ECT app for contact tracing, reporting that it was faster and more accurate to use than the paper-based system. Reported advantages of the ECT app over the paper-based system included the elimination of the CTC’s travel time to receive the daily lists of contacts from the District, the ability to monitor accurately large numbers of contacts, and the relative ease of transporting a phone compared with carrying paper forms. Suggested improvements related to using the app included the need for better network coverage (outside the control of the study or even the Ministry of Health and Sanitation); improved battery life and quality of phones; the need for further training on synchronising the data (“syncing”) between the phone and the server; the need for increased compensation to offset phone charging fees (due to decreased battery life of the donated second–hand phone batteries due to some CTCs and CTs charging their phones using alternative methods of removing and charging the phone battery only using ‘charging pegs’ at telecentres (charging booths) instead of taking the entire phone for charging); better strategies for overcoming distances to charging booths; and more refresher training for contact tracing and monitoring using the study phones.

## Discussion

To our knowledge, this is the first study to attempt to estimate the time to first contact visit from confirmation of an Ebola case for contact tracing using an app- compared with paper-based system. Our findings demonstrate proof-of-concept that an electronic system for data capture and management can improve data completeness and accuracy of Ebola contact tracing and monitoring through improved data completeness and monitoring. However, the study also illustrated the challenges of conducting high-quality contact tracing in an emergency setting [2, 5, 9, 34].

Previous studies comparing paper-based methods of data collection with electronic methods have tended to show the electronic method was superior [17, 35]. Our study showed that advantages of the app over the paper-based system included a more accurate, timely, and permanent record of information, and improved data completeness, quality and security. The challenges in collecting high-quality data using a paper-based system was shown by the poor quality of this data.

Operational requirements needed to introduce effective mobile-based contact tracing/monitoring during an Ebola epidemic include effective coordination with technical partners, understanding the structure of the evolving contact tracing and monitoring process in real-time, accessing study hardware and software, and the technical expertise to design, develop, pilot and deploy the app during the epidemic. The results are applicable to other low-income settings that face similar geographical and infrastructural conditions.

One key finding was the long interval between Ebola case and contact registration and first visit by the CT when using the ECT app. This was, on average, longer than the interval for the paper-based cases that predated the introduction of the app, though the data are not fully comparable and may be partly due to the highly-clustered nature of Ebola cases and their contacts, in space and time, with the addition of new contacts several days after the initial laboratory confirmation of the case increasing the duration between registration and first visit. The need to adhere to national protocols meant that a parallel approach of using both paper and the app was in place, which may have affected the efficiency and accuracy of the app-based method. The duration could have potentially been shortened through technical improvements in the system (e.g. batch assignment of contacts rather than single assignment of each contact by a CTC to one or more CTs), but these were impractical due to the underlying technical structure of the app. Other causes reflect structural limitations of mHealth approaches in certain settings (including poor network coverage).

### Strengths and limitations of the ECT app-based system

A key challenge was the complexity of designing and implementing an app-based system in an emergency context. Few studies have attempted to design and evaluate a mHealth intervention during an epidemic. It was necessary to minimise any risk to the existing paper-based system, and steps taken to mitigate this risk included the phased introduction of the ECT app and continued use of the paper-based DRF alongside the app as recommended by technical partners.

The major strength of the app-based system was the creation of a more accurate, timely, and permanent record of information stored in a centralised data management and data storage system in the main CommCare HQ. The system allowed for the real-time transfer of information to CTCs and CTs once the Ebola case and contacts were registered at the District level. The app-based system had in-built validation and mandatory data entry fields to improve data entry and data accuracy. Other strengths of the app-based system were the reduction in data entry work, improved data security, data sharing, and monitoring of CTC and CT activity with the production of key performance data. For example, the paper-based system meant CTs had to carry large volumes of paper to monitor contacts. Further, additional contacts could be easily added to the app-based system and assigned for contact tracing. This reduced the need for travel by CTCs to the DHMT offices to collect the Ebola contact line list, which was the daily process for the standard paper-based system. Findings from the qualitative results found that CTCs and CTs preferred to use the app over the paper-based system for contact tracing.

We were unable to test our original trial hypothesis that the app reduced the time from confirmation-to-visit by the CT as the data quality for the paper-based system was inadequate for this estimation. However, the duration for some app-based steps were longer than anticipated, reflected the multiple challenges (Table 3). For example, the time from confirmation-to-registration on the ECT app (median 18 h) reflected the standard protocol whereby the surveillance teams had to revisit the home of the Ebola case to review and, where necessary, revise the contact list after case confirmation and subsequent delays in receiving the new updated list of contacts back to the district level. This was also meant to occur for the paper-based system; however, this protocol was not always followed.

One challenge in using the app was assignment of long lists of Ebola contacts. This was due to a technical feature related to the app that resulted in errors in assignment that potentially contributed to a time delay. Further issues included the time-consuming assignment of long lists of Ebola contacts to multiple CTs, some of whom did not have their ECT app-installed smartphones switched on, or who may having forgotten how to use the app if there was a long delay between assignments. Steps to mitigate this included giving CTs pocket-sized instructions on key steps on the use of the app, field support from a study monitor and refresher training.

The average number of Ebola contacts for the paper-based system was lower than that for the app-based system (16 contacts per case compared with 36 contacts per case). The paper-based contact line listing was undertaken before contact tracing started, so the difference in the average number of Ebola contacts per case is unlikely to be due to the method used, but may reflect the varying number of contacts of Ebola cases over time and the high degree of clustering in time and space. Further, surveillance activities were enhanced in the district in June, which may have increased the number of contacts identified per case. The increased number of contacts per case during the app-system period may also reflect the increased duration from registration-to-first-visit by the CTs for the app-based versus paper-based system. The majority of cases with longer confirmation-to-visit times were from Chiefdoms with multiple contacts per case and this may have affected the process e.g. through manual errors in assigning contacts and system errors with monitoring features.

Other challenges included poor network coverage and technical challenges (e.g. corrupted memory cards and software errors), short battery life and charging issues and the time lag between the training and emergence of real Ebola cases and contacts for monitoring. Mitigating action included testing the phones before deployment and providing CTCs and CTs with spare phones/batteries, power banks for charging and changing the network provider (Additional file 1: Table S1).

Developing an app during an epidemic had additional challenges, including the speed with which the app needed to be developed, limited time for pre- and pilot testing due to the pressure to respond quickly to the emergency and difficulties in updating app design and technical features after deployment. The complex design structure of the contact tracing system had to be reflected in the technical design of the app platform. Mitigating action to overcome these issues included simulated testing of Ebola cases and contacts to test the system, further refresher training and training and the development of strategies to reduce issues related to the difficulties with the app design (some of which are listed in Additional file 1: Table S1).

### Strengths and limitations of the paper-based system

The main strength of the paper-based system was the use of a simple method of data collection. However, our study was motivated by recognised challenges in this system, including in-built delays such as the physical collection of forms from the DHMT each morning (a round trip of approximately 5–6 h depending on the Chiefdom). The geographical challenges particular to Port Loko District and the challenges for surveillance activities have been reported by others involved in surveillance activities in the District [36].

Our study identified major problems with i) data archiving of the standard paper-based system, with the majority of contact tracing forms not being returned to the DHMT at the end of the monitoring period; and ii) data quality, with missing and inaccurate information. However, contact tracing may have sometimes been conducted more efficiently than the recorded data implied - for example, direct reports by phone circumventing the paper-based system. This may have improved the time-to-first-visit using the paper-based system but not improved the app-based system as the timings for the app-based system were automatically generated.

### Recommendations to improve contact tracing

i) Addressing human resource issues is key especially the dissatisfaction with the level of financial reimbursement for the role, travel to often remote locations and hostility from local communities. These personnel challenges have been reported in other studies on Ebola contact tracing [2, 5, 7–10, 36].

ii) Improved monitoring and evaluation including stronger technical oversight of contact tracing activities and improved monitoring of CTs in particular to monitor their contact tracing activities and improved strategies to obtain DRFs, for example, setting specific days that DRFs should be returned to the District, and incentives/rewards for timely and complete data submission.

iii) For the paper-based system, we recommend that future improvements should address operational delivery issues of the paper-based system including, improved filing, data archiving and data entry systems, and greater co-ordination between organisations involved.

iv) For the app-based system, a two-tier system from registration at the District to visitation by the CT, rather than our three-tier structure would have been preferable, as the long delays were often between contact assignment to first visit. Our monitoring showed that the assignment stage presented major problems, even though CTCs were trained and received refresher training on this stage. One of the considerable constraints of the app-based system was trying to match to a paper-based system. A complex system created with paper and voice communication in mind can be difficult to replicate using the consistent logic required by software.

Other recommendations would include use of an in-country app design team where possible, further support on the technical aspects of the design, and high-quality (not second-hand) phones.

## Conclusion

This study illustrates the complexities, challenges and opportunities of implementing an app-based system for epidemic contact tracing and monitoring. Despite these challenges, this proof-of-concept study shows that implementing mHealth applications even in a low-connectivity and low-resource environment like rural Sierra Leone is possible, even in an emergency. Having more time to develop and test the app and conduct training workshops in a non-emergency setting should result in a more comprehensive and successful app that could be further integrated with the national health system.

## Additional files

Additional file 1: **Table S1**. Key monitoring issues/challenges identified with the ECT app. (DOCX 26 kb)

Additional file 2: The Ebola Contact Tracing (ECT) app. (PDF 644 kb)

## Data Availability

The datasets analysed during the current study are available in the LSHTM data repository https://datacompass.lshtm.ac.uk/1069/

## Abbreviations

App: Application
CDC: Centers for Disease Control
CIT: Case Investigation Team
CT: Contact Tracer
CTCs: Contact Tracing Coordinators
CTs: Contact Tracers
DERC: District Ebola Response Centre
DHMT: District Health Management Team
DRF: Daily Reporting Form
ECT app: Ebola Contact Tracing application
ECT: Ebola Contact Tracing
FGDs: Focus Group Discussions
GIZ: Deutsche Gesellschaft für Internationale Zusammenarbeit GmbH
GPS: Global Positioning System
IDs: Identifications
IMC: International Medical Corps
IPA: Innovations for Poverty Action
IQR: Interquartile Range
LSHTM: London School of Hygiene & Tropical Medicine
mHealth: Mobile Health
PHE: Public Health England
RSLAF: Republic of Sierra Leone Armed Forces
SD: Secure Digital
SIM: Subscriber Identity Card
SL: Sierra Leone
SLESRC: Sierra Leone Ethics and Scientific Review Committee
SMS: Short Message Service
SQ: Saira Qureshi
TB: Tuberculosis
UNFPA: United Nations Population Fund
UNMEER: United Nations Mission for Ebola Emergency Response
US: United States
WHO: World Health Organization

## Footnotes

1 A listed contact is a contact who was line listed by the case investigation team

2 Secret burials are burials that were conducted without the knowledge of the health authorities and those involved in the safe management of dead bodies and burials of patients who died from suspected or confirmed Ebola virus disease. Deceased individuals buried through secret burials were often buried by family/friends and community members and were therefore not tested for Ebola virus disease.
