# Supplementary material for: Use of a mobile application for Ebola contact tracing and monitoring in northern Sierra Leone: a proof-of-concept study
Source: BMC Infect Dis. 2019 Sep 18;19:810. doi: 10.1186/s12879-019-4354-z (PMC6749711; doi:10.1186/s12879-019-4354-z)
Supplement: Supplementary file 1 — Table S1. Key monitoring issues/challenges identified with the ECT app. (DOCX 26 kb) [file 12879_2019_4354_MOESM1_ESM.docx]

|  | | |
| --- | --- | --- |
| **Type of issue** | **Key monitoring issues/challenges observed** | **Mitigation strategies** |
| Technical – hardware | - Technical issues with study phones (e.g. short battery life, hardware issues) - Use of second-hand donated phones (several phones had technical issues when tested) | - Testing of all study phones before deployment/spare study phones, batteries and SD cards in all Chiefdoms - New phone batteries procured |
| Technical – general issues | - Mobile network coverage issues causing syncing issues with the app - Main study phone screen freezing problems - SD memory card corruption error messages - Phone battery recharging issues due to often distance to tele-centres to charge study phones - Short phone battery life/depleted batteries due to use of universal chargers at telecentres - Phone operation problems e.g. CTCs and CTs experiencing problems with switching on mobile data/logging into CommCare following training | - Change of phone network provider - Phone restart instructions - Reinstallation of CommCare and app Module/Replacement of study phone - Spare/replacement batteries issued to all CTCs and CTs |
| Technical software - ECT app Registration stage | - Technical issues on the display of Ebola case and contact information after registration (disappearing information from Registration Module) - Non-functioning edit feature for Ebola case and contact information (reported to the technical team – difficult to rectify) - Non-functioning symptomatic and non-visited contacts feature in the past 24 hours (reported to technical team – difficult to rectify) | - Monitoring using a separate electronic tablet at the District to view information on Ebola case and contacts at the assignment stage - Direct editing of Ebola case and contact information in the main CommCareHQ. - Continued use of existing daily reporting mechanisms to the district through calls to the GOAL Contact Tracing Manager by CTCs and reporting guidelines for symptomatic contacts |
| Technical –software- ECT app – Assignment stage) | - Incorrect assignment of contacts by CTCs e.g. choosing the named Chiefdom that appeared under the list of CTs to assign to - Re-assignment of contacts by other CTC of contacts - Incorrect sequencing/logic of contact numbering viewing for assignment by CTC - Syncing issues due to phone network and technical phone problem caused delays in assigning contacts - Non-functioning symptomatic contacts and non-visited contacts feature in the past 24 hours requiring manual checking in main CommCare HQ/reliance on phone calls to alert about symptomatic contacts - Few instances of no mobile data available eg mobile data had been used up | - Study protocols and procedures notes/training guides for CTCs - Support from monitor/study team to reassign Ebola contacts - Refresher training (August 2015) on assignment steps and assigning Ebola contacts given sequencing logic issues - Monitoring of assigned contacts at District level by the Data Coordinator - Bulk mobile data transfer and immediate transfer of data for those whose mobile data ran out |
| Technical software – ECT app – Visitation stage | - Syncing issues to receive Ebola contact information - Poor phone network coverage/CTs forgetting to sync information after monitoring – information not received in the main CommCare HQ - Difficulties obtaining GPS location reading led to depleted phone battery - Initial CommCare main page log-in problems - Difficulties with the CTs remembering steps to use the study phone and conduct the monitoring visit due to non-use of the phone for real monitoring e.g. lack of real Ebola cases occurring in particular Chiefdoms | - Field team support/troubleshooting/ replacement study phone issued - SIM phone network changed/Field support/troubleshooting - Field support/troubleshooting/Field procedure manual/Manual skip of GPS stage - Portable credit card size instructions and instruction on CommCare log on procedures and syncing - Additional support from CTC/Field team/study helpline - Active monitoring from the study team |
| Monitoring & field issues | - Assignment to inactive CTs e.g. who lived far distances from Ebola contacts and assignment to many CTs - Delays/difficulties meeting CTC within the Chiefdom/District to reassign - Contact tracers not syncing phones to receive Ebola contacts assigned to them - Non-reporting of damaged/stolen study phones and problems with study phones - Difficulties with real time reporting issues of daily updates to the District due to phone network, syncing and battery issues - Long contact line listings and difficulty meeting with CTC to reassign correctly when Ebola contacts were incorrectly assigned - Assignment by the CTC to multiple CTs some of whom were often inactive or had forgotten how to conduct monitoring/use the study phones - Additional contact monitoring of non-quarantined homes by CTCs and CTs led to increased workload – low priority for app use - Tampering with study phones/removal of SD cards at the charging telecentre led to phone software problems - Simultaneous use of paper Daily Reporting Form in some cases led to use of the app not being prioritised | - Support from Field monitor/study team to reassign Ebola contacts - Refresher training on assignment steps for CTCs - Study protocols and procedures notes/training guides issued - Support from monitor/study team on monitoring process - Portable credit card size instructions on app monitoring steps - Study helpline to support CTCs and CTs - Support from study field team |

**Table S1: Key monitoring issues/challenges identified with the ECT app**
