# Supplementary material for: Use of a mobile application for Ebola contact tracing and monitoring in northern Sierra Leone: a proof-of-concept study
Source: BMC Infect Dis. 2019 Sep 18;19:810. doi: 10.1186/s12879-019-4354-z (PMC6749711; doi:10.1186/s12879-019-4354-z)
Supplement: Supplementary file 2 — The Ebola Contact Tracing (ECT) app. (PDF 644 kb) [file 12879_2019_4354_MOESM2_ESM.pdf]

The Ebola Contact Tracing (ECT) app

The app had three modules plus an SMS-based alert system (Table 1; Figures 2 - 16):

1) Registration: Following laboratory-confirmation, the case and their contacts were registered on the ECT app by trained DHMT staff using an electronic SIM-enabled tablet at the DHMT Data Office (Figures 2 and 3).

2) Assignment: CTCs (one per Chiefdom) assigned the contacts registered for each confirmed Ebola case to one or more CTs in their Chiefdom (Figures 4 – 10).

3) Visitation: The CTs entered data on the ECT app during the daily monitoring visit to a contact. This was then synchronized to a central server as soon as a data connection over a mobile phone network was available. This applied to all entered data: registration, assignment and visitation (Figures 10 - 16).

4) Telerivet alert: This system was designed to alert the DHMT to symptomatic contacts identified using the ECT app through an automated SMS dispatch built into the CommCare platform. The DHMT offices had consistent internet connectivity and could always send and receive information from the system.

ECT app registration stage

The ECT app registration stage is the stage at which the confirmed Ebola case and their contacts were registered on the app at the district by the data coordinator.

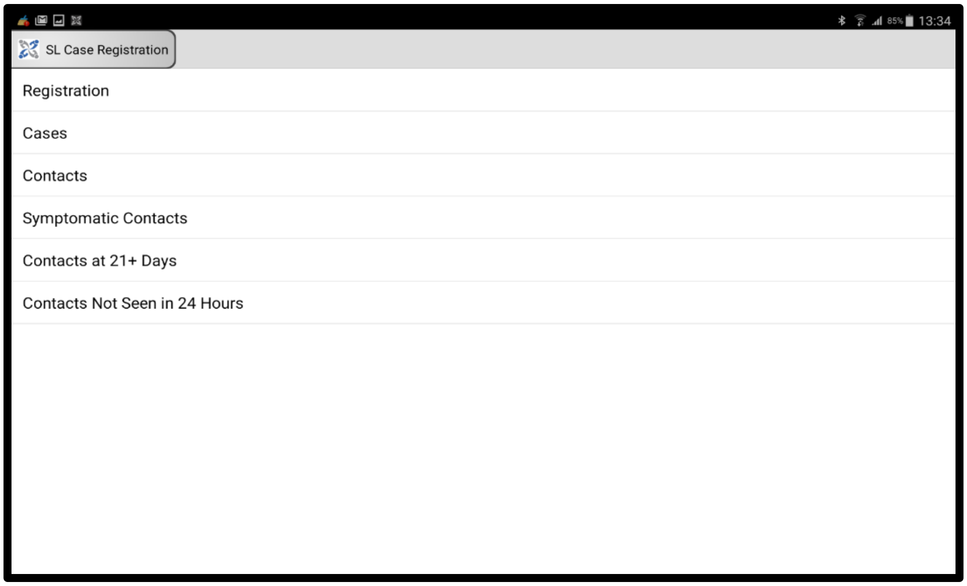

Figure 2: Sierra Leone (SL) Ebola case registration – Main summary screen

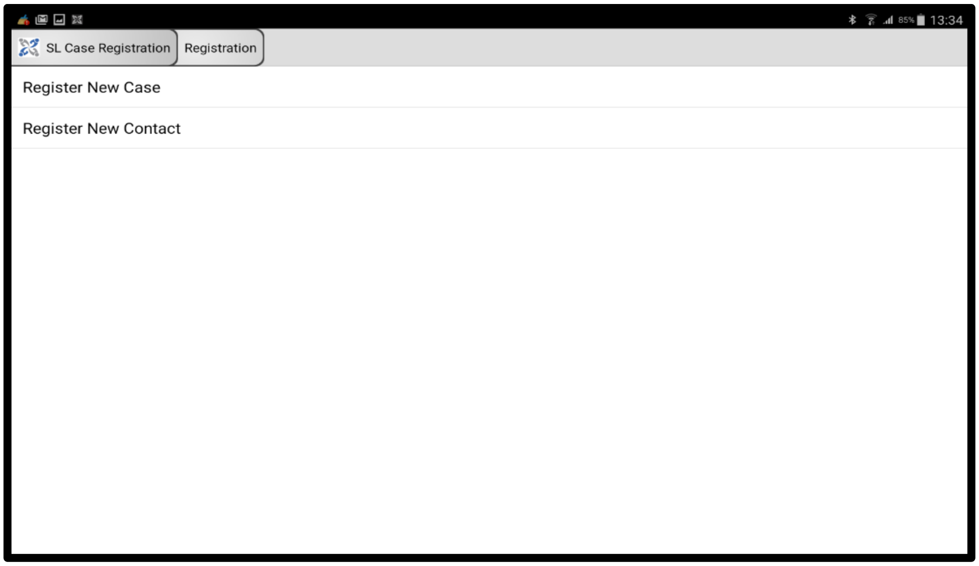

Figure 3: SL Ebola case registration – Registration screen

ECT Assignment stage app

The ECT Assignment stage was the stage at which the contacts of the Ebola cases that were registered at the registration stage were assigned by contact tracing coordinators to a contact tracer(s) in their Chiefdom.

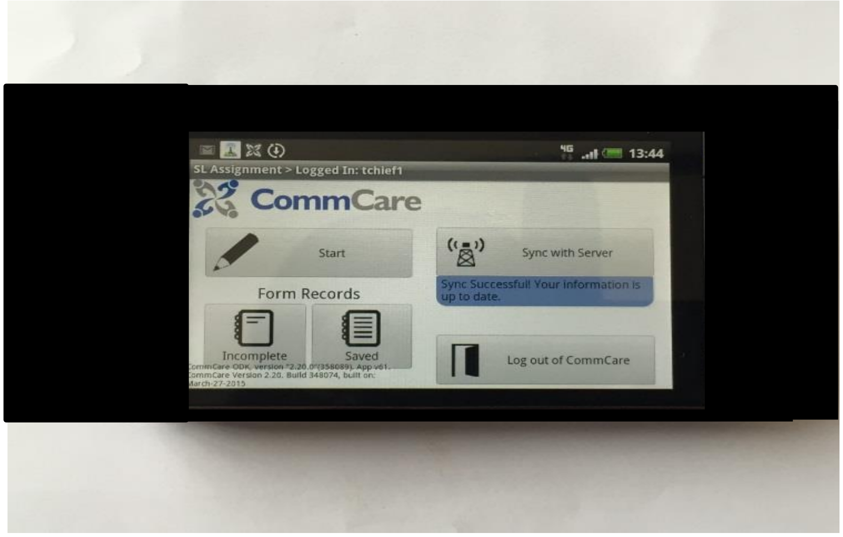

Figure 4: SL Assignment module-Start screen

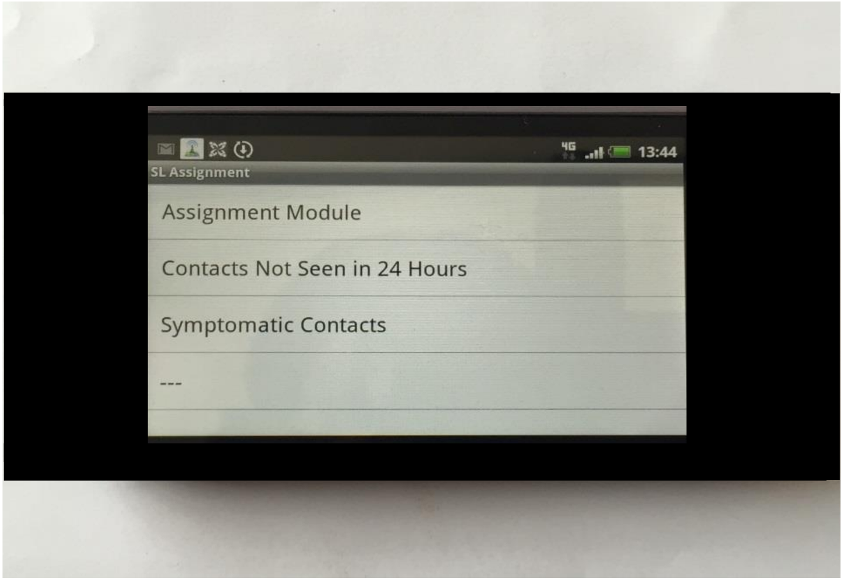

Figure 5: SL Assignment Module – Selection screen

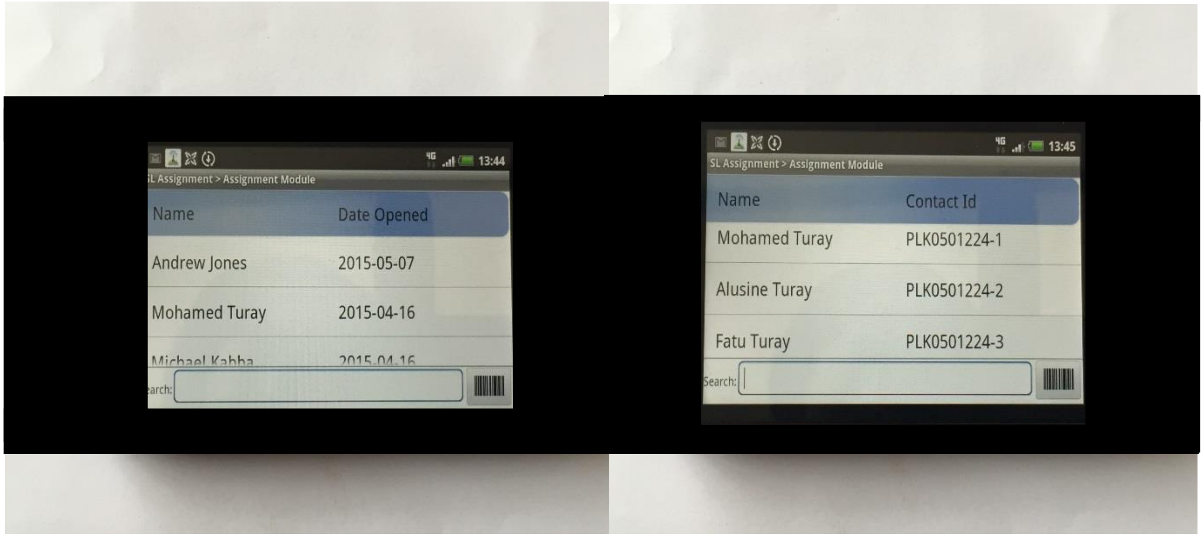

Figures 6 and 7: SL Assignment Module – Ebola case display screen (fictitious case – Figure 6) and contacts of fictitious case (Figure 7)

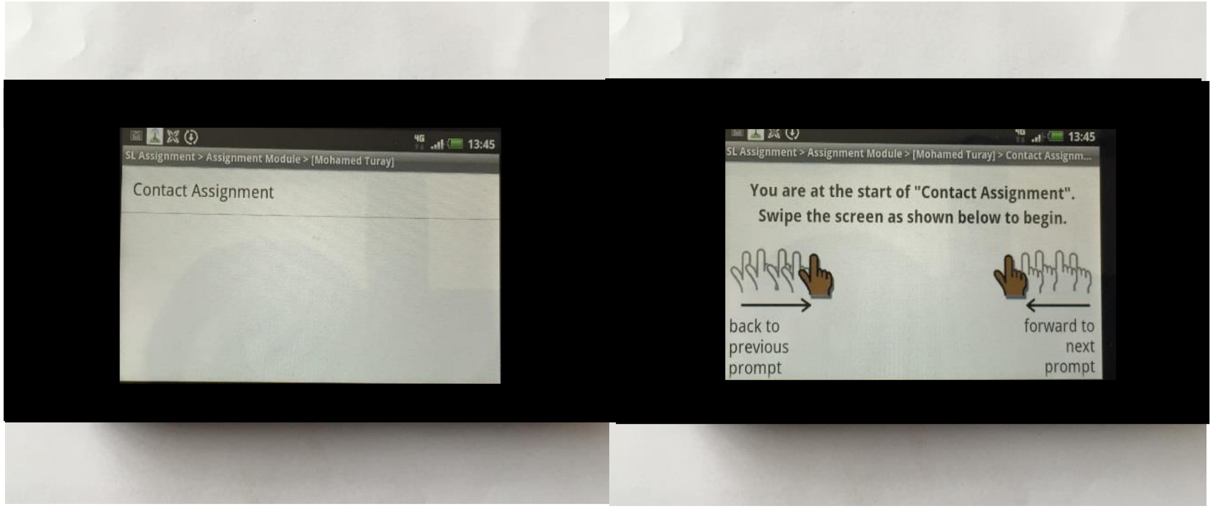

Figures 8 and 9: SL Assignment Module – Contact assignment screen and start of Contact assignment screen

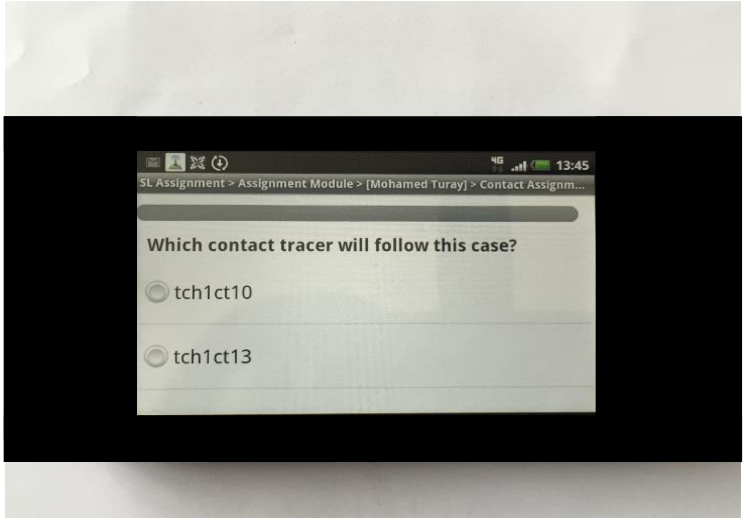

Figure 10: SL Assignment Module – Contact assignment screen (note: real contact tracer names were used for the main study version of the ECT app)

ECT Visitation stage app

The ECT Visitation stage of the app was the stage in which the contact tracer used the app to monitor the Ebola contact for 21 days. Features including whether the Ebola contact was well or sick, and if they were, sick, what their symptoms were. Additional information including the GPS location of the Ebola contact was also recorded.

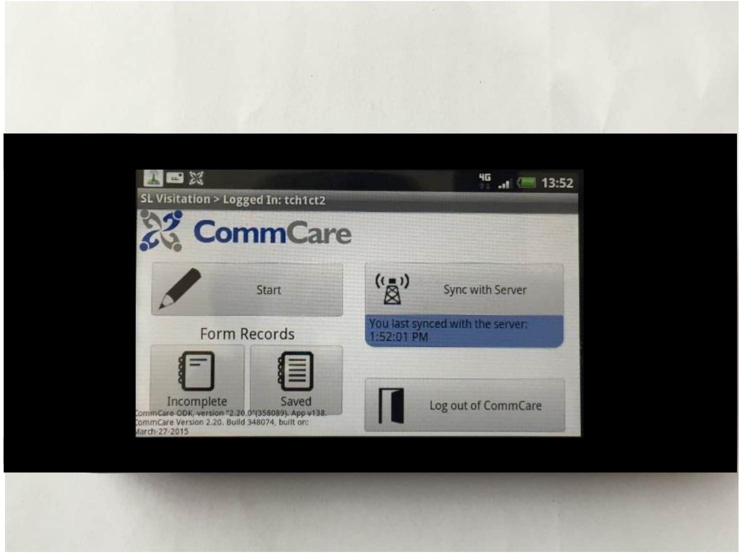

Figure 11 : SL Visitation Module – Start screen

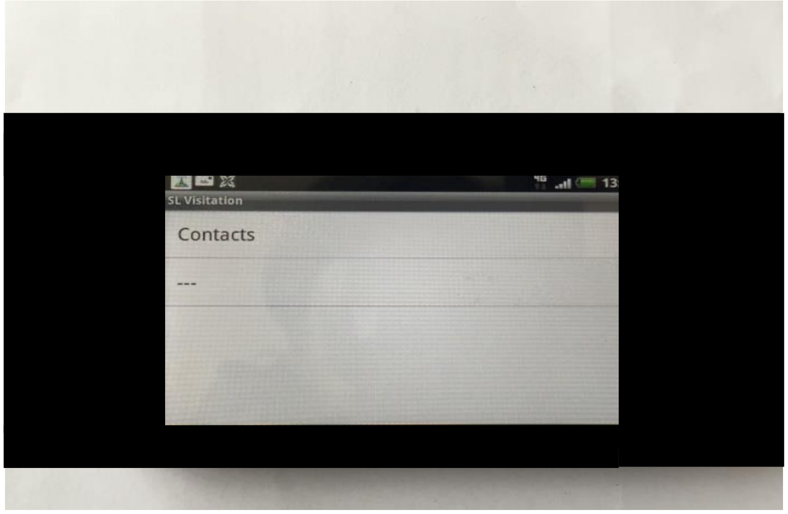

Figures 12: SL Visitation Module – Contacts screen

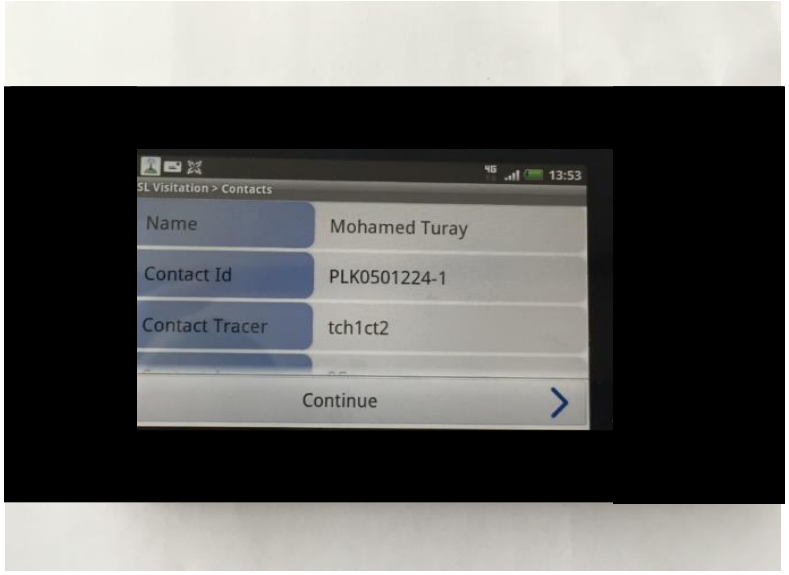

Figures 13: SL Visitation Module – Contact information screen

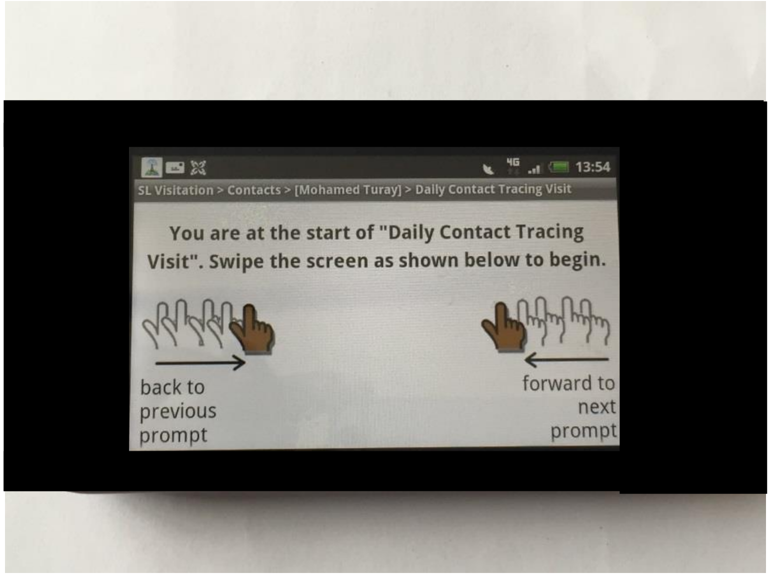

Figure 14: SL Visitation – Start of Daily contact tracing visit screen

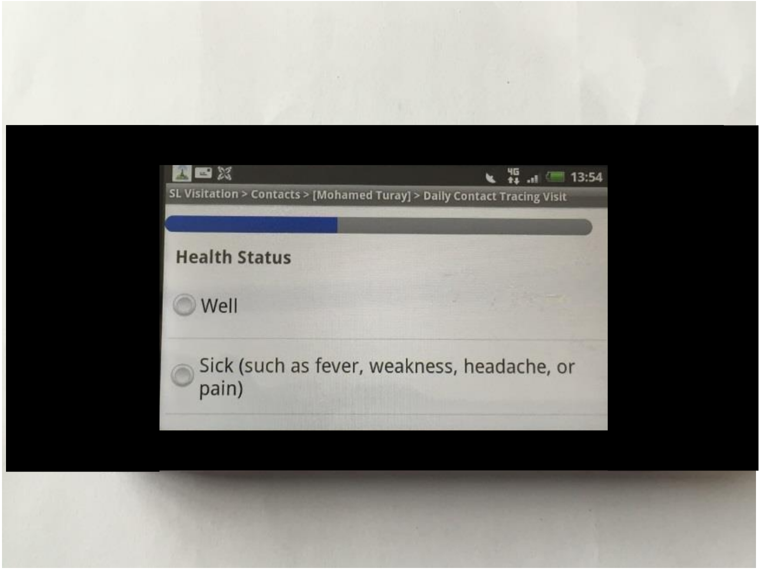

Figure 15: SL Visitation Module – Health status of contact screen

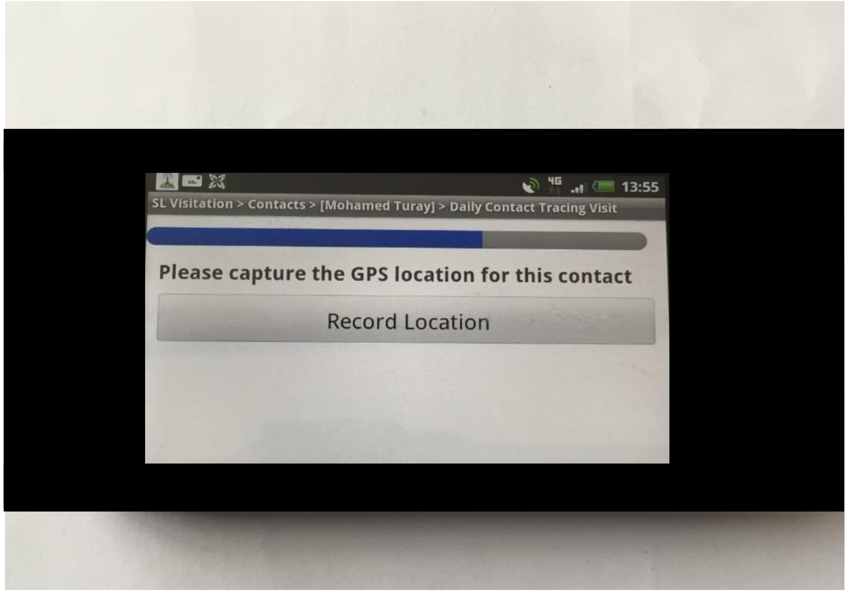

Figure 16: SL Visitation Module – Record GPS location of contact screen
